# The burden of low back pain in Brazil: estimates from the Global Burden of Disease 2017 Study

**DOI:** 10.1186/s12963-020-00205-4

**Published:** 2020-09-30

**Authors:** Caroline Nespolo de David, Lucas de Melo Castro Deligne, Rodolfo Souza da Silva, Deborah Carvalho Malta, Bruce B. Duncan, Valéria Maria de Azeredo Passos, Ewerton Cousin

**Affiliations:** 1 Postgraduate Program in Epidemiology, Universidade Federal do Rio Grande do Sul, Rua Ramiro Barcelos, 2600 Sala 414, Bairro Santa Cecilia, Porto Alegre, 90035-003 Brazil; 2 Hospital de Clínicas de Porto Alegre, Porto Alegre, Rio Grande do Sul Brazil; 3 Faculdade Ciências Médicas de Minas Gerais, Belo Horizonte, Brazil; 4 Telehealth Centre, Universidade Federal do Rio Grande do Sul, Porto Alegre, Brazil; 5 Department of Maternal and Child Nursing and Public Health, Nursing School, Universidade Federal de Minas Gerais, Belo Horizonte, Brazil

**Keywords:** Low back pain, Burden of disease, Prevalence, Years lived with disability, Disability-adjusted life years

## Abstract

**Background:**

The prevalence and burden of musculoskeletal (MSK) conditions are growing around the world, and low back pain (LBP) is the most significant of the five defined MSK disorders in the Global Burden of Disease (GBD) study. LBP has been the leading cause of non-fatal health loss for the last three decades. The objective of this study is to describe the current status and trends of the burden due to LBP in Brazil based on information drawn from the GBD 2017 study.

**Methods:**

We estimated prevalence and years lived with disability (YLDs) for LBP by Brazilian federative units, sex, age group, and age-standardized between 1990 and 2017 and conducted a decomposition analysis of changes in age- and sex-specific YLD rates attributable to total population growth and population ageing for the purpose of understanding the drivers of changes in LBP YLDs rates in Brazil. Furthermore, we analyzed the changes in disability-adjusted life years (DALYs) rankings for this disease over the period.

**Results:**

The results show high prevalence and burden of LBP in Brazil. LBP prevalence increased 26.83% (95% UI 23.08 to 30.41) from 1990 to 2017. This MSK condition represents the most important cause of YLDs in Brazil, where the increase in burden is mainly related to increase in population size and ageing. The LBP age-standardized YLDs rate are similar among Brazilian federative units. LBP ranks in the top three causes of DALYs in Brazil, even though it does not contribute to mortality.

**Conclusions:**

Findings from this study show LBP to be the most important cause of YLDs and the 3rd leading cause of DALYs in Brazil. The Brazilian population is ageing, and the country has been experiencing a rapid epidemiological transition, which generates an increasing number of people who need chronic care. In this scenario, more attention should be paid to the burden of non-fatal health conditions.

## Background

Epidemiological transitions correlate with an increase in life expectancy of the population, a decrease in deaths due to maternal and infectious diseases and an increase in chronic diseases—not only related to mortality but also to disability [1, 2]. An aging population is expected to dramatically increase the burden of musculoskeletal (MSK) conditions over the coming decades [3]. Data from the Global Burden of Disease (GBD) study quantified the significant burden of MSK disorders, and its recent publications have shown that the prevalence and burden of MSK conditions are growing around the world [4–6]. The five defined MSK disorders in the GBD study are low back pain (LBP), neck pain, osteoarthritis, rheumatoid arthritis, and gout. LBP is the most significant of these, ranking as the top cause of years lived with disability (YLDs) in GBD 2017 MSK disorders —especially in the high-income, high-middle-income, and middle-income countries (as defined by the socio–demographic index)—and has been the leading cause of non-fatal health loss for the last three decades [6]. A recent series of publications reinforce warnings about the increasing magnitude of this problem [7–10].

Data from a Brazilian national survey in 2003 indicate back pain as the most reported chronic disease, affecting 13.2% of the adult population [11]. In a 2013 national health survey, 18.5% of the Brazilian population reported chronic back pain [12]. There are also reports that back pain is among the main factors contributing to absence from work and early retirement in Brazil [13]. Leading factors associated with chronic back pain in Brazil were older age, low education level, female gender, history of smoking, heavy physical activity at work or at home, and being overweight or obese [12, 14].

LBP is a complex condition with multiple contributors to both the pain and the associated disability, including psychological factors, social factors, biophysical factors, comorbidities, and pain-processing mechanisms [7]. There are common symptoms affecting all age groups from children to the elderly population [15]. The high frequency, chronicity, and resultant disability of LBP impose a considerable economic burden. The financial impact is cross sectoral because it increases costs in both health care and social support systems. LBP is among the most prevalent causes of absence from work and medical consultations worldwide [16, 17]. However, this disorder has not been on focus of public health programs, especially in low-income and middle-income countries [18].

Although studies of LBP and associated factors have been undertaken in Brazil, the burden of this disease has not yet been well described. The objective of this study is to describe the current status and trends of the burden due to LBP in Brazil based on information drawn from the GBD 2017 study.

## Methods

This is a descriptive study using estimates from the Global Burden of Diseases (GBD) 2017 study, coordinated by the Institute for Health Metrics and Evaluation (IHME) at the University of Washington. Data are available in the GBD results tool [19] and the GBD compare data visualization [20]. GBD estimates the disease burden for 359 diseases and injuries, from 1990 to 2017, covering 195 countries and territories. A detailed description of the robust methodological approach used in the GBD analysis has been published previously [6, 21–23]. Briefly, GBD uses three main indicators to calculate disease burden—years of life lost due to premature mortality (YLLs), years lived with disability (YLDs), and the sum of YLLs with YLDs: disability-adjusted life years (DALYs) [24]. As there is no mortality from LBP, YLD and DALY estimates are the same.

The input data for LBP in Brazil was mainly 12 studies, covering the period from 2002 to 2013, most of which was conducted in the state of Rio Grande do Sul. The full list of articles used for statistical modeling can be found in the GBD 2017 data input sources tool [25]. To estimate prevalence, incidence, and YLDs, the Bayesian meta-regression tool DisMod-MR 2.1 is used [26]. YLDs are calculated by multiplying the sequela of each condition prevalence by the disability weight, a value attributed for the health state of each sequela condition. In addition, correction for comorbidity is performed through the COMO (comorbidity correction) tool.

LBP is defined as low back pain (with or without pain referred into one or both lower limbs) that lasts for at least 1 day a year [6]. The “low back” is defined as the area on the posterior aspect of the body from the lower margin of the 12th ribs to the lower gluteal folds [6]. The ICD-10 codes for LBP are M54.3, M54.4, and M54.5, and the ICD-9 code is 724 [6]. LBP is not a cause of death, as it is an exclusively disabling condition. The disability weights, used in the YLDs estimation, are measured on a scale from 0 to 1, with 0 as a state equivalent to full health and 1 equivalent to death. Briefly, disability weights were obtained from surveys conducted in several countries from different regions. The surveys used paired comparison questions, in which respondents considered two hypothetical individuals with different, randomly selected health states and indicated which person they regarded as healthier [27, 28]. More methodologic detail on their application in calculating disability is available elsewhere [6]. Table 1 from supplementary material describes LBP sequelae classifications and the health state with its disability weight.

For this study, we generated the estimates of LBP prevalence and YLDs by Brazilian federative units, sex, and age group between 1990 and 2017. To help understand the drivers of changes in LBP YLDs rates in this period, we conducted a decomposition analysis of changes due to total population growth, population aging, and changes in age- and sex-specific YLD rates for Brazil, following methodology previously used in the GBD project, based on Das Gupta [16]. In addition, we analyzed the position and changes in LBP YLDs and DALYs ranking in Brazil over the same period.

To describe the degree of confidence in the indicators and take account of uncertainties in the initial data and subsequent calculations, GBD generates 95% uncertainty intervals (95% UI) for its metrics, as described elsewhere [6]. Results were expressed for all ages or were age standardized for the world population [29].

The Global Burden of Disease Study Brazil 2017 was approved by the Ethics Committee of Federal University of Minas Gerais (UFMG) under registration no. 628033167.00005149, according to the resolution no. 466/2012 of the Brazilian National Health Council.

## Results

### Prevalence

**Fig. 1:**
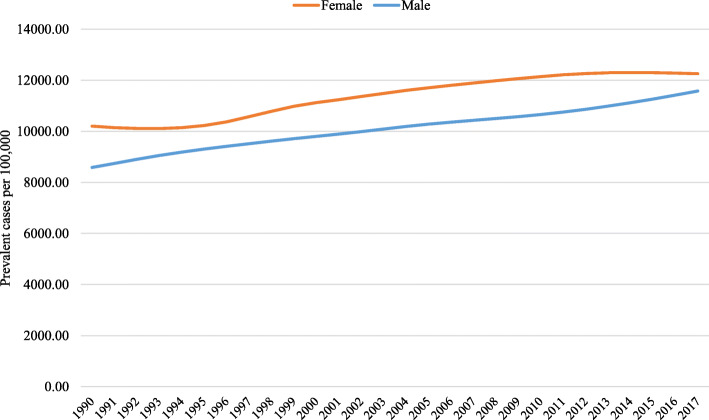
Brazilian low back pain prevalence rate from 1990 to 2017 for all ages by sex

According to GBD estimates, in 2017, approximately 25 million Brazilians had LBP, with a prevalence rate per 100,000 inhabitants of 11,924.78 (95% UI 10,622.04–13,301.04), representing an increase of 26.83% (95% UI 23.08 to 30.41) from the prevalence rate observed in 1990 (9402.31; 95% UI 8336.82–10,558.93). Figure 1 shows the prevalence rate of LBP between 1990 and 2017 for all ages by sex in Brazil. For women, LBP prevalence rate increased by 20.14% (95% UI 16.18 to 23.87), from 10,201.89 (95% UI 9041.71–11,422.26) in 1990 to 12,256.54 (95% UI 10,941.21–13,646.53%) in 2017, and the higher increase occurred between 1995 and 2010. For men, LBP prevalence rate increased by 34.86% (95% UI 30.61 to 39.04), from 8584.69 (95% UI 7609.92–9656.17) in 1990 to 11,577.69 (95% UI 10,269.28–12,997.60) in 2017, and increased occurred throughout the period. The prevalence increases with age, and it may reach 21,762.12 (95% UI 17,776.23–26,025.14) among people aged 70 years and over (data not shown).

Brazilian LBP prevalence rate for both sexes, all ages by federal units in 2017 are present in Table 1. The state with highest all ages prevalence rates is Rio Grande do Sul (13,370.04 per 100,000; 95% UI 12,023.48-14,874.39). Amapá had the lowest all ages prevalence rate, 10,104.26 (95% UI 8931.84-11,462.49). The southern and southeastern states tend to have a higher LBP prevalence than the northern and northeast states. The difference between the state with the highest and the lowest prevalence was 32%.

**Table 1:** All ages and age-standardized prevalence, and YLDs rates in Brazil, in 2017 and percentage change from 1990 to 2017

Location	All-age prevalence rate (per 100,000)			All-age YLD rate (per 100,000)			Age-standardized YLD rate (per 100,000)		
	1990	2017	Percentage change, 1990-2017	1990	2017	Percentage change, 1990-2017	1990	2017	Percentage change, 1990-2017
Global	6997.50 (6231.79 to 7780.02)	7551.65 (6792.00 to 8339.52)	7.92% (5.60 to 9.90)	788.23 (559.37 to 1060.75)	850.04 (608.76 to 1144.13)	7.84% (5.51 to 9.95)	892.27 (636.62 to 1195.47)	810.34 (582.41 to 1089.10)	−9.18% (−10.30 to − 8.06)
Brazil	9402.31 (8336.82 to 10,558.93)	11,924.78 (10,622.04 to 13,301.04)	26.83% (23.08 to 30.41)	1061.50 (755.03 to 1455.27)	1348.96 (961.44 to 1823.61)	27.08% (23.20 to 30.74)	1243.04 (890.26 to 1686.11)	1243.58 (888.04 to 1681.16)	0.04% (−1.84 to 1.91)
Acre	7998.42 (7102.00 to 8995.64)	10,547.14 (9280.34 to 11,963.75)	31.87% (26.18 to 37.78)	899.89 (639.76 to 1226.37)	1190.95 (841.72 to 1640.12)	32.34% (26.25 to 38.41)	1241.58 (886.00 to 1678.31)	1255.54 (890.35 to 1701.50)	1.12% (**−**3.17 to 5.77)
Alagoas	8395.19 (7424.57 to 9446.36)	10,891.15 (9677.66 to 12,236.10)	29.73% (24.00 to 35.78)	943.35 (663.23 to 1284.26)	1230.55 (886.49 to 1673.82)	30.44% (24.19 to 37.04)	1223.83 (870.80 to 1650.85)	1235.78 (891.78 to 1672.26)	0.98% (**−**3.47 to 5.48)
Amapá	7488.47 (6609.51 to 8475.88)	10,104.26 (8931.84 to 11,462.49)	34.93% (28.80 to 41.25)	846.12 (593.54 to 1168.46)	1147.11 (816.58 to 1575.11)	35.57% (28.86 to 42.38)	1191.12 (842.37 to 1610.39)	1209.68 (863.44 to 1639.97)	1.56% (**−**2.74 to 5.96)
Amazonas	7909.17 (6939.83 to 8920.56)	10,430.54 (9247.56 to 11,734.43)	31.88% (25.65 to 38.41)	893.53 (629.38 to 1236.54)	1183.96 (846.68 to 1611.99)	32.50% (26.02 to 39.69)	1230.85 (880.77 to 1668.51)	1252.08 (890.67 to 1706.85)	1.72% (**−**2.31 to 6.34)
Bahia	8509.18 (7552.90 to 9523.50)	11,378.85 (10,105.44 to 12,689.92)	33.72% (27.44 to 40.07)	958.66 (679.37 to 1315.57)	1286.80 (916.26 to 1756.09)	34.23% (27.29 to 41.40)	1225.32 (873.18 to 1654.67)	1237.24 (878.52 to 1686.38)	0.97% (**−**3.34 to 5.04)
Ceará	8843.70 (7887.93 to 9903.95)	11,322.03 (9982.37 to 12,697.56)	28.02% (22.69 to 33.93)	996.49 (707.23 to 1355.14)	1279.81 (916.02 to 1726.37)	28.43% (22.88 to 34.58)	1226.19 (877.90 to 1654.69)	1235.96 (882.73 to 1667.00)	0.80% (**−**3.39 to 5.15)
Distrito Federal	8921.79 (7845.27 to 10,144.68)	11,812.81 (10,452.66 to 13,282.73)	32.40% (25.22 to 40.05)	1012.50 (714.54 to 1410.84)	1344.82 (951.14 to 1829.70)	32.82% (25.59 to 41.27)	1239.88 (885.59 to 1695.84)	1257.08 (896.75 to 1696.25)	1.39% (**−**2.9 to 5.66)
Espírito Santo	9022.63 (7950.13 to 10,144.27)	11,906.41 (10,563.55 to 13,275.72)	31.96% (24.9 to 38.95)	1016.72 (720.61 to 1385.71)	1346.96 (959.54 to 1825.51)	32.48% (25.13 to 40.15)	1207.58 (865.97 to 1637.66)	1221.88 (868.26 to 1649.34)	1.18% (**−**3.68 to 6.01)
Goiás	9155.85 (8053.32 to 10,367.38)	12,061.59 (10,713.64 to 13,565.84)	31.74% (24.89 to 38.98)	1035.09 (733.38 to 1417.14)	1366.18 (972.11 to 1859.18)	31.99% (24.74 to 39.34)	1237.09 (882.58 to 1675.13)	1253.08 (892.46 to 1699.99)	1.29% (**−**3.32 to 5.91)
Maranhão	8020.64 (7097.91 to 9042.25)	10,331.02 (9180.42 to 11,575.16)	28.81% (23.26 to 34.45)	901.24 (642.62 to 1231.51)	1165.62 (827.16 to 1571.69)	29.34% (23.66 to 35.47)	1222.12 (875.10 to 1662.09)	1236.29 (876.76 to 1659.13)	1.16% (**−**3.24 to 5.83)
Mato Grosso	8609.23 (7609.64 to 9763.26)	11,692.27 (10,348.35 to 13,115.60)	35.81% (27.96 to 44.09)	974.83 (686.89 to 1335.28)	1326.96 (938.82 to 1828.63)	36.12% (28.14 to 44.73)	1236.56 (881.35 to 1674.35)	1255.37 (893.49 to 1712.96)	1.52% (**−**2.97 to 6.11)
Mato Grosso do Sul	9114.06 (8000.83 to 10,284.81)	12,036.61 (10,691.01 to 13,476.10)	32.07% (25.35 to 39.02)	1029.83 (727.66 to 1412.35)	1359.04 (975.95 to 1849.51)	31.97% (24.92 to 39.51)	1244.28 (886.38 to 1691.48)	1260.84 (903.01 to 1707.70)	1.33% (**−**3.01 to 5.95)
Minas Gerais	9545.36 (8513.09 to 10,756.95)	12,535.36 (11,121.03 to 14,012.25)	31.32% (24.53 to 38.59)	1078.39 (768.07 to 1474.84)	1418.74 (1007.94 to 1920.07)	31.56% (24.62 to 38.97)	1246.63 (889.07 to 1700.14)	1259.37 (892.67 to 1705.65)	1.02% (**−**3.28 to 5.48)
Pará	8048.34 (7122.44 to 9074.84)	10,501.53 (9286.45 to 11,758.56)	30.48% (24.61 to 36.30)	907.85 (642.28 to 1234.54)	1189.31 (834.78 to 1619.83)	31.00% (24.72 to 37.31)	1212.20 (865.33 to 1641.37)	1232.64 (871.04 to 1663.29)	1.69% (2.47 to 6.18)
Paraíba	9128.90 (8097.37 to 10,182.79)	11,474.20 (10,192.56 to 12,847.02)	25.69% (19.82 to 31.07)	1026.66 (733.85 to 1390.82)	1295.82 (928.33 to 1770.20)	26.22% (19.98 to 31.85)	1236.40 (884.51 to 1686.17)	1241.76 (887.79 to 1697.14)	0.43% (**−**4.00 to 4.71)
Paraná	9618.16 (8475.91 to 10,804.47)	12,539.12 (11,138.33 to 14,033.40)	30.37% (23.69 to 36.91)	1088.05 (770.49 to 1489.98)	1419.44 (1014.47 to 1915.32)	30.46% (23.35 to 37.65)	1251.66 (894.84 to 1701.67)	1260.79 (901.84 to 1713.37)	0.73% (**−**3.44 to 5.10)
Pernambuco	8941.29 (7904.24 to 10,039.26)	11,337.99 (10,058.43 to 12,714.66)	26.80% (21.10 to 32.83)	1005.99 (719.89 to 1373.08)	1280.39 (911.97 to 1738.82)	27.28% (21.07 to 33.67)	1218.01 (879.70 to 1653.55)	1231.87 (877.21 to 1671.30)	1.14% (**−**3.26 to 5.59)
Piauí	8527.90 (7562.74 to 9595.49)	11,349.02 (10,132.17 to 12,720.15)	33.08% (26.70 to 40.03)	961.75 (686.19 to 1304.56)	1283.10 (912.90 to 1731.95)	33.41% (26.25 to 40.98)	1246.66 (896.78 to 1687.65)	1254.96 (892.42 to 1695.32)	0.67% (**−**3.76 to 5.41)
Rio de Janeiro	10,940.05 (9556.56 to 12,296.12)	11,766.97 (10,353.58 to 13,375.62)	7.56% (1.52 to 13.43)	1236.71 (883.40 to 1697.27)	1332.54 (947.04 to 1815.16)	7.75% (1.39 to 14.11)	1296.01 (930.10 to 1758.69)	1159.64 (815.81 to 1582.42)	**−**10.52% (**−**14.82 to **−**5.86)
Rio Grande do Norte	8911.47 (7883.71 to 9987.35)	11,456.63 (10,194.50 to 12,790.66)	28.56% (22.43 to 34.87)	1004.63 (711.46 to 1363.24)	1295.26 (923.13 to 1745.17)	28.93% (22.53 to 35.85)	1226.10 (879.40 to 1660.54)	1234.50 (882.33 to 1665.16)	0.69% (**−**3.55 to 5.02)
Rio Grande do Sul	10,332.16 (9261.45 to 11,600.95)	13,370.04 (12,023.48 to 14,874.39)	29.40% (22.86 to 36.59)	1164.60 (821.69 to 1577.12)	1504.27 (1070.59 to 2028.55)	29.17% (22.05 to 36.86)	1249.78(885.67 to 1679.18)	1300.04 (927.98 to 1750.78)	4.02% (**−**1.04 to 10.15)
Rondônia	8306.92 (7302.47 to 9452.61)	11,608.96 (10,294.07 to 13,097.69)	39.75% (31.93 to 47.59)	937.95 (659.48 to 1287.93)	1315.61 (934.36 to 1802.36)	40.26% (32.12 to 48.46)	1233.07 (881.74 to 1674.41)	1256.89 (892.66 to 1704.12)	1.93% (**−**2.33 to 6.54)
Roraima	8055.68 (7045.15 to 9176.25)	10,350.25 (9129.27 to 11,702.51)	28.48% (21.24 to 35.81)	912.33 (637.70 to 1269.23)	1173.62 (829.32 to 1599.26)	28.64% (21.24 to 35.81)	1224.99 (876.08 to 1664.32)	1249.97 (886.85 to 1678.71)	2.04% (**−**2.15 to 6.26)
Santa Catarina	9500.02 (8377.49 to 10,703.65)	12,392.52 (10,948.38 to 14,012.16)	30.45% (23.05 to 37.57)	1075.95 (762.65 to 1464.24)	1404.03 (1000.90 to 1915.42)	30.49% (22.72 to 38.12)	1232.70 (879.50 to 1664.21)	1239.37 (886.55 to 1685.83)	0.54% (**−**4.57 to 5.25)
São Paulo	9968.04 (8771.07 to 11,232.13)	12,630.62 (11,206.54 to 14,185.03)	26.71% (20.50 to 32.72)	1127.16 (803.51 to 1542.47)	1429.44 (1018.97 to 1936.00)	26.82% (20.26 to 33.31)	1244.08 (882.39 to 1672.60)	1258.85 (895.74 to 1693.95)	1.19% (**−**3.25 to 5.91)
Sergipe	8481.56 (7486.18 to 9517.51)	11,160.54 (9880.95 to 12,520.99)	31.59% (25.64 to 38.10)	954.03 (673.76 to 1299.43)	1262.54 (905.14 to 1707.08)	32.34% (25.64 to 39.24)	1214.45 (872.41 to 1648.33)	1227.26 (879.39 to 1643.98)	1.06% (**−**3.21 to 5.47)
Tocantins	8356.80 (7394.14 to 9416.92)	11,199.22 (9931.18 to 12,608.95)	34.01% (28.31 to 39.97)	942.62 (666.65 to 1293.52)	1266.16 (901.33 to 1716.69)	34.32% (28.19 to 41.21)	1236.53 (883.26 to 1667.80)	1255.61 (894.66 to 1695.16)	1.54% (**−**2.84 to 5.74)

### Burden

**Fig. 2:**
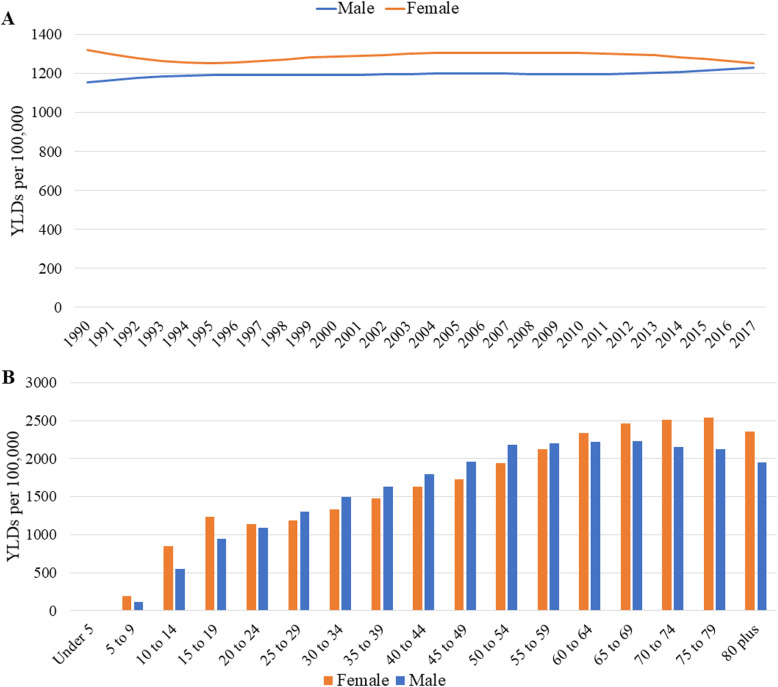
Low back pain YLDs rates per 100,000 by sex in Brazil, 2017. **a** Age-standardized YLDs per 100,000. **b** YLDS per 100,00 by age groups

In Brazil, there were 2,857,276.66 YLDs due to LBP in 2017, up to 80% from the 1,586,096.66 YLDs in 1990. The age-standardized YLDs rates attributable to LBP between 1990 and 2017 had smaller changes: in women, there was a 5% (95% UI −7.73 to −2.40) decrease from 1322.22 YLDs (95% UI 943.55–1790.30) per 100,000 in 1990 to 1255.89 YLDs (95% UI 897.80–1691.06) per 100,00 in 2017, and in men, there was a 6.27% (95% UI 4.10 to 8.37) increase from 1157.15 YLDs (95% UI 826.96–1568.43) per 100,000 in 1990 to 1229.68 YLDs (95% UI 876.64–1668.23) per 100,00 in 2017 (Fig. 2a). YLDs rates for LBP have growth between the ages of 5–9 to 65–69 years, reaching 2353.91 YLDs (95% UI 1483.59–3529.19) per 100,000. The ages at which the YLD burden is greater between 75-79 years (Fig. 2b). After this age, the LBP burden reduces slightly.

**Fig. 3:**
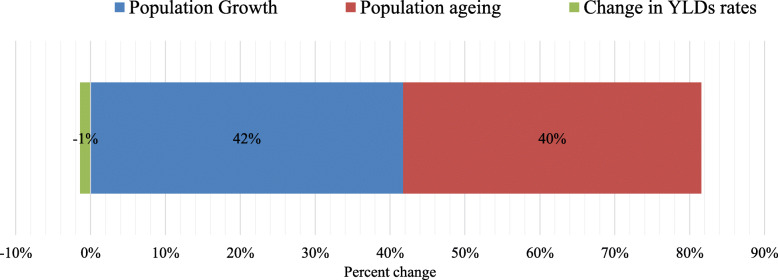
Decomposition analysis of LBP YLDs (thousands) change from 1990 to 2017

Figure 3 shows the decomposition analysis of the change of YLDs in Brazil due to LBP between 1990 and 2017. Population growth accounted for an increase of 42%, aging of the population accounted for an increase of 40%, and changes in the underlying age- and sex-standardized rates of YLDs accounted for a 1% decrease in the absolute number of YLDs due specifically to LBP over the period.

According to Table 1, southern and southeastern states tend to have higher LBP YLD rates, and northern states tend to have lower values. The all-ages YLDs rates in Brazil by federative units show a higher burden in the states of Rio Grande do Sul, São Paulo, and Paraná, and a lower all ages YLDs rates in Amapá, Maranhão, and Roraima. Nonetheless, when YLDs rates are age-standardized, the estimates are similar between the states. Rio Grande do Sul is the state with the highest age-standardized burden of LBP, with 1300.04 (95% UI 927.98-1750.78) YLDs per 100,000, and Rio de Janeiro is the state with the lowest age-standardized YLDs rate, 1159.64 (95% UI 815.81-1582.42) per 100,000, and the relative difference between them is 12%. The percentage change in age-standardized YLDs rate from 1990 to 2017 was similar in almost all states, Rio Grande do Sul had the highest increase, 4.02% (95% UI −1.04 to 10.15), and Rio de Janeiro was the only one who showed a reduction in the period (−10.52% 95% UI −14.82 to −5.86). Additionally, the difference between the Brazilian state with the lowest rate of YLDs age standardized, Rio de Janeiro, and the global estimates (810.34 YLDs per 100,000; 95% UI 582.41−1089.10) is 43% (Table 1).

**Fig. 4:**
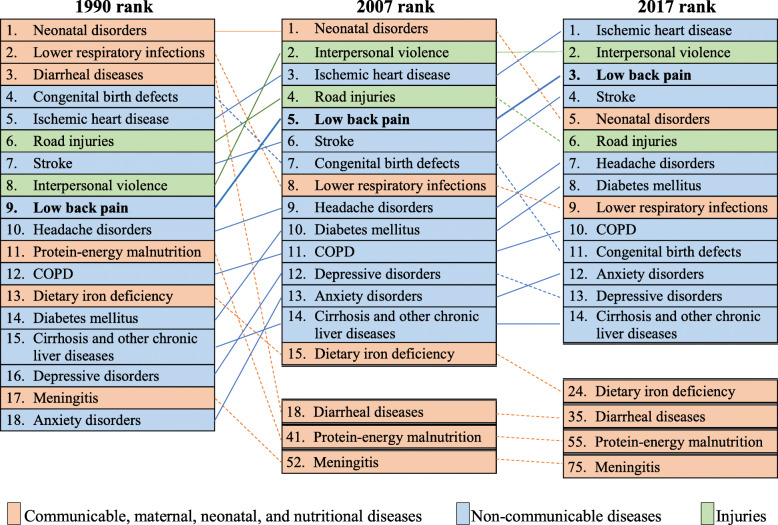
DALYs rank rate per 100,000 (both sexes and all ages) in Brazil, 1990–2007–2017. COPD, chronic obstructive pulmonary disease

In Brazil, LBP was the major cause of disability in 2017, the age-standardized rate was 1243.58 (95% UI 888.04-1,681.16) YLDs/100,000. For comparison, it is 38% higher than the second cause, headache disorders (898.54, 95% UI 587.46-1287.30). Brazilian LBP DALYs ranking for both sexes, all ages, went from the ninth to fifth position between 1990 and 2007, and ranked third position in GBD diseases in 2017. Figure 4 shows this change and allows comparing the position of LBP in relation to other diseases that contribute significantly to mortality rates; for example, it is ranked above causes such as stroke and diabetes.

## Discussion

This study highlights the high prevalence and burden of LBP in Brazil. We found that LBP prevalence increased by 26.82% (95% UI 23.08 to 30.41) between 1990 and 2017 and tends to be higher among southern and southeastern Brazilian states. However, it appears to be increasing more between northern, northeastern, and midwestern federative units in the last 27 years. LBP represents the most important cause of YLDs in Brazil, remaining in the first place of rankings of YLDs for GBD diseases in this 27-year period. Although the increase in this burden is mainly related to increases in population size and aging. As with prevalence, GBD also estimated higher rates of YLDs for the southern and southeastern states, but the increase in the period 1990-2017 is similar in almost all states, except for Rio de Janeiro that had a decrease. Brazilian age-standardized YLDs rates are similar between states and higher than global estimates. LBP is a disease that does not lead to death. Hence, it does not contribute to years of life lost (YLL), and 100% of all LBP DALYs are due to YLDs. The chronicity of LBP and the fact that it begins at younger ages causes the burden for LBP to rank in the top three causes of DALYs in Brazil.

The Brazilian National Health Survey (PNS) 2013 found a prevalence of back pain of 18.5% in persons 18 years or older, higher than GBD estimates [12]. The PNS evaluated chronic pain with the question: “Do you have a chronic back problem, such as chronic back or neck pain, LBP, sciatica, vertebrae or disc problems?” but did not delimit the period of the pain occurrence, while the GBD assessed prevalence in last year (at least 1 day in last 12 months) only for LBP [12]. This difference in classifications could in part explain the differences between GBD prevalence estimates and PNS. Furthermore, results from the PNS were not included in the data selection process used to generate GBD estimates; that is, the real values may be even larger, which only reinforces the magnitude of the problem.

Although in this study the prevalence rate of LBP seems to be higher among women, there was no statistically significant difference in relation to men’s rates. A higher prevalence of LBP in women has been observed in some previous studies [12, 30]. In the literature, this has been attributed to the greater awareness of women about the symptoms and signs of diseases [14, 31]; factors such as performing housework in greater intensity in non-ergonomic position and repetitive tasks [31]; differences in anatomical and functional characteristics, such as smaller height, less muscle mass, less bone mass, joints more fragile, and less adapted to strenuous physical effort, may result in more overload in the back [31]; pregnancy factors, like hormones, weight increase, inadequate posture during pregnancy, postural inadequacies when breastfeeding, the child’s weight, and other factors [31].

Age is one of the most common risk factors for MSK conditions. The ratio of older to younger people will continue to increase throughout the world. Additionally, the number of people who are obese, which is now one of the major risk factors for MSK conditions, is expected to increase more dramatically in low-income and middle-income countries over the coming two decades [8]. Data from a Brazilian national survey shows that factors associated with a higher prevalence of chronic back pain in both sexes, adjusted by age and education, were increasing age; low education level; smoking history; high salt intake; heavy activity at work or at home, and the increase in the time spent on these activities; being overweight or obese; having chronic diseases; and poor health assessments [12]. The main risk factors for LBP according to the GBD study are occupational ergonomic factors, smoking, and high body mass index [26].

LBP is the largest cause of MSK disability in Brazil [6] and a large proportion of those affected are in their most productive years of life. This can have a major effect on a family’s livelihood as the ability to be productive in these years is often required to support younger and older family members [3]. Chronic back pain is one of the most commonly reported complaints by the adult population, causing disability, reduced functionality, absence from work, and the most common cause of social security pension disability claims and early retirements in Brazil [13]. In addition to indirect costs, related to lower productivity, time away from work, and expenses resulting from sick leaves and early retirements, the direct costs are quite significant—tests, medications, physical therapy, and hospitalizations [31]. Between 1995 and 2014, expenditure on spinal surgery in Brazil increased by 540% (from R$27.1 million to R$146.5 million) [8]. Nevertheless, few studies exist for these disabling health conditions.

Progress in reducing the impact of disabling conditions has been much slower—the focus remains on reducing mortality rather than the main causes of disability. The slower progress in addressing non-fatal compared with fatal health outcomes and aging of populations make YLDs an increasingly important component of global DALYs. In some countries with advanced aging, YLDs already make up more than half of the total burden in DALYs [6].

The growing number of people affected by MSK conditions in low- and middle-income countries, including Brazil, is of great concern. Health services are not prepared to meet such demand. A paradigm shift is urgently needed if we are to alleviate the increasing global burden of non-fatal diseases, such as MSK conditions, and reduce the number of avoidable disabilities [3].

Our findings call for the integration of prevention and control programs for MSK disorders within health system programs, which may reduce the severity of disabilities. Interventions should include control of known risk factors especially through health education and awareness; ergonomic factors in occupational health and safety assessments; provide evidence-based early diagnosis and treatment; rehabilitative care and community programs to increase knowledge of relevant risk and protective factors.

A recent meta-analysis of population-based interventions to prevent LBP concluded that combined strengthening with stretching or aerobic exercises two to three times per week can be recommended to prevent LBP [32]. Greater exercise appears to prevent incident episodes of LBP, and the effect may be greater when exercise is combined with self-care education [33]. Greater effort by the Brazilian National Health System (SUS: Sistema Único de Saúde) and private health systems to stimulate the regular practice of physical activity and stretching should be pursued. The relative lack of studies on the prevention and management of this condition also calls for a greater priority in research funding for investigation of the prevention and management of LBP. In addition, it is necessary to improve the collection of health data to monitor trends and efficacy of interventions.

There are inherent limitations in estimating LBP, because their measurement is fully dependent on self-reported metrics and the recognition of the disease by the individual depends on the degree of perception, frequency of signs and symptoms. To mitigate this, the GBD 2017 study makes adjustments for variations in recall period, anatomical location, minimum duration of episodes, and the extent to which the condition limits activity. Also, there are limitations inherent to the GBD process estimation. One of them is the absence of studies about LBP prevalence in all Brazilian states. Rio Grande do Sul was the state which presented the highest rates for prevalence and YLDs for LBP, and also was the one with the largest number of studies. It is possible that estimates may be under assessed in other states. To mitigate this other limitation, GBD uses complex modeling to get closer to real estimates.

## Conclusion

Findings from this study show LBP to be the most important cause of YLDs and the 3rd leading cause of DALYs in Brazil. The Brazilian population is aging, and the country has been experiencing a rapid epidemiological transition, which generates an increasing number of people who need chronic care. In this scenario, more attention should be paid to the burden of non-fatal health outcomes.

## Supplementary information

**Additional file 1:** **Supplementary Table 1.** Sequelae, health states, health state lay descriptions, and disability weights for low back pain, GBD 2017.

## Data Availability

The data used are publicly available online on the website of the Institute for Health Metrics and Evaluation (IHME) (http://ghdx.healthdata.org/gbd-results-tool)

## Abbreviations

DALYs: Disability-adjusted life years
GBD: Global Burden of Disease
IHME: Institute for Health Metrics and Evaluation
LBP: Low back pain
MSK: Musculoskeletal
PNS: Brazilian National Health Survey
YLDs: Years lived with disability
YLLs: Years of life lost
