# Supplementary material for: The burden of low back pain in Brazil: estimates from the Global Burden of Disease 2017 Study
Source: Popul Health Metr. 2020 Sep 30;18(Suppl 1):12. doi: 10.1186/s12963-020-00205-4 (PMC7526352; doi:10.1186/s12963-020-00205-4)
Supplement: Supplementary file 1 — Additional file 1: Supplementary Table 1. Sequelae, health states, health state lay descriptions, and disability weights for low back pain, GBD 2017. [file 12963_2020_205_MOESM1_ESM.docx]

**Supplementary material**

Supplementary table 1 – Sequelae, health states, health state lay descriptions, and disability weights for low back pain, GBD 2017.

| **Sequela** | **Health state name** | **Health state lay description** | **Disability weight (UI)** |
| --- | --- | --- | --- |
| Most severe low back pain without leg pain | Back pain, most severe, without leg pain | has constant back pain, which causes difficulty dressing, sitting, standing, walking, and lifting things. The person sleeps poorly, is worried, and has lost some enjoyment in life. | 0.372 (0.250-0.506) |
| Severe low back pain without leg pain | Back pain, severe, without leg pain | has severe back pain, which causes difficulty dressing, sitting, standing, walking, and lifting things. The person sleeps poorly and feels worried. | 0.272 (0.182-0.373) |
| Most severe low back pain with leg pain | Back pain, most severe, with leg pain | has constant back and leg pain, which causes difficulty dressing, sitting, standing, walking, and lifting things. The person sleeps poorly, is worried, and has lost some enjoyment in life. | 0.384 (0.256-0.518) |
| Mild low back pain with leg pain | Mild low back pain with leg pain | (combined DW) | 0.020 (0.011-0.035) |
| Mild low back pain without leg pain | Low back pain, mild | has mild back pain, which causes some difficulty dressing, standing, and lifting things. | 0.020 (0.011-0.035) |
| Moderate low back pain without leg pain | Low back pain, moderate | has moderate back pain, which causes difficulty dressing, sitting, standing, walking, and lifting things. | 0.054 (0.035-0.079) |
| Severe low back pain with leg pain | Back pain, severe, with leg pain | has severe back and leg pain, which causes difficulty dressing, sitting, standing, walking, and lifting things. The person sleeps poorly and feels worried. | 0.325 (0.219-0.446) |
| Moderate low back pain with leg pain | Moderate low back pain with leg pain | (combined DW) | 0.054 (0.035-0.079) |

UI: uncertainty interval; DW: disability weight

Fonte - GBD 2017 Disability Weights (1)

1. Institute for Health Metrics and Evaluation (IHME). Global Burden of Disease Study 2017 (GBD 2017) Disability Weights | GHDx. Seattle, WA: IHME, University of Washington, 2018. Available from http://ghdx.healthdata.org/record/ihme-data/gbd-2017-disability-weights. (Accessed January 10, 2019).
